# Evaluation of remote monitoring of parturition in dairy cattle as a new tool for calving management

**DOI:** 10.1186/1746-6148-9-191

**Published:** 2013-10-01

**Authors:** Claudio Palombi, Marco Paolucci, Giuseppe Stradaioli, Mario Corubolo, Paolo B Pascolo, Maurizio Monaci

**Affiliations:** 1 Department of Pathology, Diagnostic and Veterinary Clinics, University of Perugia, Via San Costanzo 4, 06126 Perugia, Italy; 2 Department of Agricultural and Environmental Sciences, University of Udine, Via delle Scienze 206, 33100 Udine, Italy; 3 Department of Civil Engineering and Architecture, University of Udine, Via delle Scienze 206, 33100 Udine, Italy

**Keywords:** Dairy cattle, Calving monitoring, Dystocia, Post-partum fertility

## Abstract

**Background:**

Proper calving management of dairy herds is a crucial aspect of the bovine life cycle, as it has profound effects on calf viability and on the post-partum course of the dam. The objectives of this study were to monitor the calving process through the use of a remote alarm system and to determine the impact of prompt emergency obstetric procedures in case of dystocia for the prevention of stillbirths and post-partum reproductive pathologies, and for improving herd fertility. Six groups of experimental animals were studied: monitored heifers (*n* = 60) and multiparous cows (*n* = 60) were compared with non-observed animals (*n* = 60 heifers and *n* = 60 multiparous) giving birth during the same time period and housed in the calving barn, and with unmonitored animals placed in a dry zone (*n* = 240 heifers and *n* = 112 multiparous cows).

**Results:**

The incidence of dystocia ranged from a minimum of 23.4% (monitored multiparous cows) to a maximum of 33.3% (monitored heifers), and there were no differences compared with control groups. However, the rate of stillbirth was higher in control groups than in the monitored groups (*P* < 0.01). Among both heifers and multiparous cows, the incidence of post-partum uterine infections was higher in the unmonitored animals both in the calving barn (*P* < 0.01) and in the dry zone (*P* < 0.05) compared with monitored animals. Among both heifers and multiparous cows, the control groups showed higher rates of foetal membrane retention than did the monitored groups (*P* < 0.001). The calving-to-conception interval was shorter; in particular, observed heifers showed a significant advantage of approximately 46 days compared with the unmonitored group (*P* < 0.001) and 32 days compared with the group in the calving barn (*P* < 0.05). Multiparous cows also had a reduction in the number of days open.

**Conclusions:**

The remote alarm system used to monitor the calving process assured the prompt presence of personnel, improving both the cow’s reproductive efficiency and neonatal viability.

## Background

Insufficient monitoring around the time of parturition in dairy cattle might prolong the birth process unnecessarily, thereby increasing the risk of both stillbirth [1-4] and calving complications associated with impaired reproductive performance leading to an increased calving-to-conception interval [3]. Another important reason further justifying the effort of monitoring the calving process is to ensure the correct morphological and functional development of the calf by means of colostrum ingestion within the first 6 h of parturition [5,6]. Ingestion of an adequate level of colostral IgG is essential to improve the health and survival of neonatal calves [7].

To predict the exact moment at which the calving process begins, various protocols have been proposed including ultrasound monitoring [8], observing changes in the body temperature [9-11], analysing blood levels of oestrone sulphate and 17-β-oestradiol [12], evaluating blood levels of progesterone [13], controlling the level of relaxation of pelvic ligaments [14], determining the concentration of electrolytes present in mammary gland secretions [15] and, lastly, video monitoring the animals [16].

Although these calving monitoring technologies have been developed, none have been adopted widely by producers, and visual observation of the cow’s behaviour is still the most frequent practical approach. Monitoring approximately every 3–6 h from the first detection of the onset of the first stage of labour is advisable to recognise the beginning of the second stage of normal calving and to detect abnormal deliveries early, but this is labour- and time-consuming; moreover, the continuous presence of an observer during stage two of calving has been associated with an increased number of calving problems and assisted deliveries [17].

In equine management, the delivery alarm system is widely used. Recently, an electronic system originally made for mares has been used in the dairy industry. This alarm, once attached to the *labia vulvaris,* is activated by the physical separation of the vulva lips, which pulls the actuating magnet from the transmitter, thus generating a radio-wave frequency signal that is transmitted to the Global System for Mobile communications (GSM). Our preliminary observations for the use of this system in dairy cows showed that the loss of calves could be minimised through emergency obstetrical assistance, thus resulting in an improvement in reproductive performance [18]. Various patents of calving alarm systems, which are based on different operating principles, have been registered. Some of these systems are already present in the market, but in the international literature, there is no data regarding the application of these alarm systems in the field.

The objectives of this study were to monitor the calving process through the use of a GSM-based remote alarm system and to determine its impact in term of the reduction of post-partum reproductive pathologies and stillbirths, and improvements in herd fertility.

## Methods

### Animals

This study was carried out at a dairy farm located in Umbria, a temperate climate region in central Italy (42°95′ N, 12°39′ E), over 4 years. In total, 984 records were available for inclusion in the analysis [19]. The healthy Holstein Friesian cows and heifers assigned to the study had free access to a total mixed ration formulated with corn silage (9.20% CP, 45.90% NDF and 27% ADF), oat hay (8.70% CP, 61.30% NDF and 38.20% ADF), wheat straw (4.60% CP, 78.90% NDF and 48.40% ADF) and concentrate (28.50% CP, 20.20% NDF and 9.70% ADF) in a ratio of 17.3:43.2:22.9:16.6, respectively (dry matter basis). A dietary supplement containing vitamins and minerals was available. Animals were divided into groups of heifers and multiparous cows, based on their parity and included in the study depending on the following criteria:

• Age at calving restricted to 22–30 months for heifers and 31–75 months for multiparous cows;

• Parity range from 1 (heifers) to 5;

• Body condition score [20] of 3.75 ± 0.50 and 3.50 ± 0.25 (mean ± SD), for heifers and multiparous cows, respectively;

• No previous reproductive pathologies (at calving and during the post-partum period).

**Figure 1:**
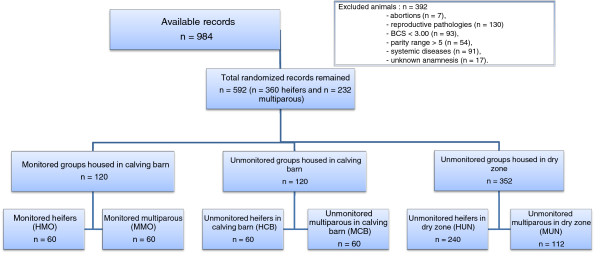
Flow chart depicting the steps used for selecting animals eligible for the study and their subdivision into groups.

To meet the above criteria, 392 animals were excluded for various reasons (Figure 1).

Only births (single or twin) from dams that were at least 50% Holstein or Friesian with no known beef breed component were retained. A total of 592 records remained (Figure 1). The experimental animals were housed in the calving barn in well-bedded individual.

### Experimental design

The experimental design included six groups (Figure 1). One group (H) was composed of heifers (n = 360) and further subdivided into three subsets, the first of which was composed of animals monitored with a GSM device (group HMO, *n* = 60), the second subset of which was not observed and gave birth in the same time period as the HMO group (group HCB, *n* = 60) and the third subset was unmonitored and housed in the dry zone with permanent litter (group HUN, *n* = 240). The second group (M) was composed of 232 multiparous cows further subdivided into three subsets, the first of which was monitored with a GSM device (group MMO, *n* = 60), the second of which was not observed and gave birth in the same time period as the MMO group (group MCB, *n* = 60), and the third subset was unmonitored and housed in the dry zone with permanent litter (group MUN, *n* = 112). Data from groups HCB, HUN, MCB and MUN served as controls; in these unmonitored animals the level of calving assistance ranged from some (from 8.00 pm till 4.00 am) to slight calving assistance alone (by one person for the rest of the day). For the entire experimental period, twice a month, the farm’s management software was used to choose animals randomly on the basis of expected calving date [19]. Only cows that calved within a range of 15 days were included in the research through random assignment to one of the groups. The animals calving in the calving barn (HMO, HCB, MMO and MCB) were paired for analysis, whereas all the others cow calving in the dry zone were included in the HUN or MUN groups based on parity. If the selected animals did not calve in the range of 15 days, they were not included in the results.

The experimental activity was carried out in accordance with the guidelines on use of animals for experimentation set by the Italian Decree Law 116/92 and has been approved by the Ethical Committee of Perugia University and the Italian Ministry of Health.

### Pre-partum evaluation and application of the intra-vaginal GSM device

**Figure 2:**
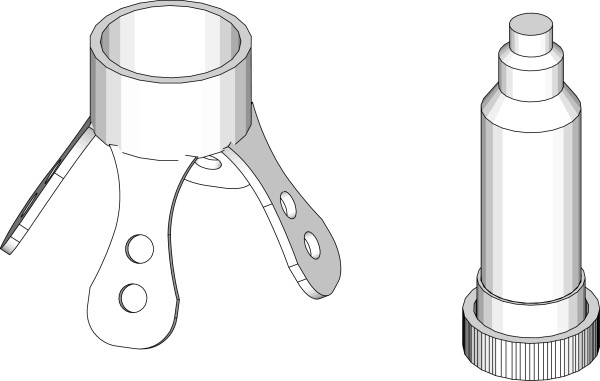
Prototype of the intra-vaginal device employed in the experiments.

Animals were moved to the calving barn when showing signs of imminent calving, such as sacrosciatic ligament relaxation, udder development, vulvar enlargement and softening and looseness of vulvar wrinkles. The prototype was inserted into the cranial portion of the vagina 3 ± 1 d before predicted calving. The system consisted of an intra-vaginal device that was pushed out at the second stage of calving and activated a radio transmitter, which in turn sent a coded signal (433 MHz) to an integrated receiver system connected to a GSM autodialer (Sinclair, Mondialtec Srl, Rome, Italy). The warning device for the detection of delivery was characterised by a probe composed of two stackable pieces: a base and a cylindrical bin (Figure 2). The base consisted of an anchoring system that secured the device to the vaginal wall, while the bin contained physical sensors and the transmitter able to signal when the device exited the animal’s body. The transmitter sent a radio signal decoded by a receiving station that activated the GSM autodialer.

The calving barn consisted of covered lots (8 × 4 m) and a separate place for the GSM receptor and transmitter placement. The receiver and autodialer were located within 30 m of the calving area. The floor of the calving barn was bedded with wheat straw replaced daily.

### Obstetrical and neonatal assistance

For control animals (groups HCB, HUN, MCB and MUN), the data relative to obstetrical and neonatal assistance were not available with accuracy because calvings were not observed systematically; therefore, the degree of calving difficulty could not be ascertained. In fact, only the generic need of calving assistance, stillbirth, foetal membrane retention (FMR) and uterine prolapse have been ascertained accurately because the majority of calvings occurred at night or was unobserved, and stall’s personnel have not been trained to recognize and record the degree of dystocia.

For the GSM monitored groups, upon radio frequency signal activation, a physical examination was performed on the parturient and foetal presentation, position and posture and the extent of cervical dilation; these data were recorded. During the expulsive phase, foetal progression was recorded as follows: vulvar protrusion of forelegs, head passage and complete expulsion.

Parturition duration, the time from alarm activation to foetal expulsion, was also recorded. According to Lombard *et al*., dystocia was defined as a delayed or difficult parturition [21]. The calving process was classified into five categories ranging from 0 to 4 based on the cause of dystocia, where degree 0 means eutocia or no assistance needed; 1 = delay of the second stage of labour; 2 = foetal malposition; 3 = foetal–pelvic disproportion; and 4 = vulvar or cervical stenosis and uterine torsion. Calving assistance was provided if there was a delay of the second stage of labour beyond 90 min from the time of alarm activation. Dystocia management was carried out according to recognised obstetrical procedures [22,23]. After a preliminary examination of the data and to define a gradual increase in difficulty of calving, category 1 was combined with 2 in a single category 1 defined as "mild dystocia" and category 3 was combined with 4 to form a new grade 2 ("severe dystocia").

The new-born calves of the HMO and MMO groups were subjected to clinical examination and to the APGAR score system on a scale from 0 to 2 and the resulting five values were then summed up to give an APGAR score between 0 and 10; these criteria are used as a mnemonic learning aid [24]. The HMO and MMO new-borns were fed colostrum within 50 ± 10 min after calving.

### Post-partum disease evaluation

During the post-partum period, data regarding the occurrence of stillbirth and reproductive diseases for all cows were extracted from the farm’s database [19]. Stillbirth was recorded in case of death of a calf just before, during, or within the first 48 h after parturition [25]. Data about post-partum diseases such as uterine infections, FMR, uterine prolapse, milk fever or ovarian cysts were recorded according to recognised definitions [23]. The term "uterine infections" comprised both metritis and endometritis [26].

### Statistical analysis

Reproductive traits such as the mean calving-to-conception interval and number of inseminations per conception for a 4-year period were extracted from the farm’s database [19]. All statistical analyses were performed using SPSS software [27] and differences were considered significant at *P* ≤ 0.05.

To evaluate differences between monitored groups (HMO *vs.* MMO), the mean time for the application of the intra-vaginal device, parturition duration and calving, and APGAR scores were compared using a univariate analysis of variance (ANOVA) model. Statistical analysis was performed for the number of artificial insemination (AI) applications per conception and the calving-to-conception interval considering as main factors the parity of cows (heifers *vs.* multiparous), the calving monitoring condition (monitored in the calving barn, unmonitored in the calving barn and unmonitored in the dry zone) and their interaction, using the following general linear model protocol:

$$y=\mu +P_{i}+M_{j}+{\text{PxM}}_{\text{ij}}+\epsilon_{\text{ijk}}$$

where:

μ = general mean

P_i_ = parity (1, primiparous; 2, multiparous)

M_j_ = calving monitoring (1, monitored; 2, unassisted in the calving barn; 3, unassisted in the dry zone)

ϵ_ijk_ = residual error

Differences between means were compared with the least significant differences procedure.

Considering that calving monitoring condition significantly affected the number of AI and calving-to-conception interval and that parity significantly influenced calving-to-conception interval, data were also compared between each of the six groups with the intent to establish the role of the variances among them using ANOVA with the Ryan–Einot–Gabriel–Welsch F test.

Differences between groups for the incidences of stillbirth, uterine infections, FMR, uterine prolapse, milk fever and ovarian cysts were evaluated using contingency tables and Pearson Chi-squared tests, comparing HMO with HCB and HUN, and MMO with MCB and MUN.

## Results

**Figure 3:**
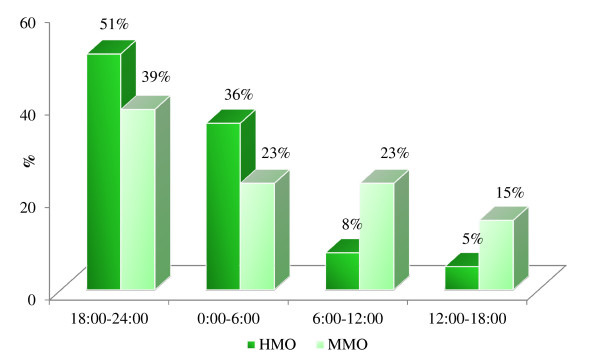
**Distribution of calving among heifers (HMO: *n*** **= 60) and multiparous (MMO: *n*** **= 60) cows within 24 h.**

The mean time needed for cleaning of each cow’s external genitalia and application of the intra-vaginal device was 5.4 ± 0.1 (mean ± SD) and 4.5 ± 0.1 min for the HMO and MMO groups, respectively (*P* **<** 0.001). Calving was observed at 36 ± 8 h following application, mainly at night (Figure 3). Every device was expelled from the vaginal canal at the second stage of calving: no false alarm and no lack of alarm when needed were recorded. Approximately 15 ± 5 min after the alarm, 68.9% of the foetuses presented with their forelegs already out of the vulvar outlet. Among the GSM-monitored animals, all calvings occurred in the dorsum-sacral position, with 113 foetuses in an anterior presentation and nine in a posterior presentation; two cases of twins were recorded. The duration of parturition was 70.7 ± 21.4 min and 59.7 ± 26.9 min for the HMO and MMO groups, respectively (*P* **<** 0.01). Calving progression in the HMO group was 44.9 ± 16.0, 60.3 ± 19.3 min and 71.4 ± 23.5 min, for passage of the forelegs, head and hind legs through the vulvar outlet, respectively. In contrast, these values in the MMO group were 36.6 ± 19.6, 39.9 ± 21.5 and 48.7 ± 27.9 min, respectively.

In the HMO group, 33.3% of the cows needed calving assistance and of these 28.3% were classified with "mild dystocia" and 5% with "severe dystocia". In the MMO group, 20% of parturitions were classified as "mild dystocia" and 3.4% as "severe dystocia", for a total of 23.4% of difficult parturitions (Table 1). Calving parameters extrapolated from the farm’s database [19] showed a similar trend of dystocia in the control groups: need of calving assistance occurred in 31.9% of the cows in the HCB group, 34.7% in the HUN group, 26.9% in the MCB group and 28.2% in the MUN group.

**Table 1:** Calving classification in the GSM-monitored animals (HMO, heifers; MMO, multiparous cows)

**Cause of dystocia**	**HMO (n = 60)**	**MMO (n = 60)**	**Calving classification**	**HMO (n = 60)**	**MMO (n = 60)**
0	–	–	Eutocia	40 (66.7%)	46 (76.6%)
1	8 (13.3%)	3 (5.0%)	Mild dystocia	17 (28.3%)	12 (20.0%)
2	9 (15.0%)	9 (15.0%)			
3	3 (5.0%)	1 (1.7%)	Severe dystocia	3 (5.0%)	2 (3.4%)
4	-	1 (1.7%)			

0 = eutocia or no assistance;

1 = delay of the second stage of labour;

2 = foetal malposition;

3 = foetal–pelvic disproportion;

4 = vulvar or cervical stenosis and uterine torsion.

The mean recorded APGAR scores were 8.6 ± 0.7 and 8.8 ± 0.8 for calves born from heifers and multiparous cows, respectively.

The prompt calving assistance provided enabled a significantly lower incidence of both puerperal and neonatal pathologies in both the HMO and MMO groups (Table 2). In particular, the MMO group was the only one with a single case of stillbirth (1.7%). Unmonitored groups in the dry zone and not observed groups in the calving barn presented with stillbirth rates ranging from 9.6 to 16.7%. The rates of stillbirth were higher in both control groups compared with the monitored group (*P* < 0.01 HUN *vs.* HMO and MUN *vs.* MMO; *P* < 0.001 HCB *vs.* HMO), except in the MCB group, which showed a trend toward significant difference from the MMO group (*P* < 0.114).

**Table 2:** Stillbirths and post-partum diseases in the six experimental groups

	**PRIMIPAROUS (n = 360)**			**MULTIPAROUS (n = 232)**		
	**Group HMO (n = 60)**	**Group HCB (n = 60)**	**Group HUN (n = 240)**	**Group MMO (n = 60)**	**Group MCB (n = 60)**	**Group MUN (n = 112)**
Stillbirth	0 (0%)	10 (16.7%)^¥^	23 (9.6%)^†^	1 (1.7%)	6 (10.0%)	16 (11.2%)^†^
Uterine infections	2 (3.3%)	14 (23.3%)^†^	34 (14.2%)^*^	2 (3.3%)	12 (20.0%)^†^	22 (19.6%)^†^
FMR	0 (0%)	10 (16.7%)^¥^	14 (5.8%)	0 (0%)	10 (16.7%)^¥^	14 (12.5%)^¥^
Uterine prolapse	0 (0%)	1 (1.7%)	2 (0.8%)	1 (1.7%)	0 (0%)	1 (0.9%)
Milk fever	1 (1.7%)	1 (1.7%)	5 (2.1%)	0 (0%)	2 (3.3%)	3 (2.7%)
Ovarian cysts	2 (3.3%)	8 (13.3%)	15 (6.2%)	1 (1.7%)	2 (3.3%)	9 (8.0%)

The HMO, HCB and HUN groups were primiparous heifers monitored by GSM, unassisted in the calving barn and unassisted in the dry zone, respectively. The MMO, MCB and MUN groups were multiparous cows monitored by GSM, unassisted in the calving barn and unassisted in the dry zone, respectively.

*FMR* foetal membrane retention.

^*^ = significantly different from the corresponding monitored group (P < 0.05);

^†^ = significantly different from the corresponding monitored group (P < 0.01);

^¥^ = significantly different from the corresponding monitored group (P < 0.001).

Among heifers, the incidence of post-partum uterine infections was significantly higher in both the HCB (*P* < 0.01) and HUN (*P* < 0.05) groups compared with the monitored animals. Multiparous cows also benefited from calving assistance, as both the MCB and MUN groups presented higher incidences of uterine infections (*P* < 0.01) than did the MMO group.

In the monitored groups, foetal membrane expulsion occurred spontaneously without any case of retention; conversely, FMR rates were higher both in the unmonitored group in the calving barn and in the not observed group in the dry zone. In particular, 10 out of 60 heifers in the HCB group (*P* < 0.001) and 14 out of 240 in the HUN group suffered membrane retention. Among multiparous cows, both the MCB and MUN groups showed significantly higher (*P* < 0.001) FMR rates than the MMO group.

The monitoring of calving led to a significantly shorter calving-to-conception interval of 115.0 days among the monitored animals compared with 143.9 and 150.5 days for cows unassisted in the calving barn and unassisted in the dry zone, respectively (Table 3). In particular, animals in the HMO group showed a significantly shorter calving-to-conception interval (46.5 days) compared with the HUN group (P < 0.001) and a 32-day shorter interval compared with the HCB group (*P* < 0.05). A similar trend was observed in the MMO group, with a duration of 26 days (*P* < 0.062) compared with the MCB group and 24 days (P < 0.066) compared with the MUN group.

**Table 3:** Mean number (± SD) of inseminations per conception and calving to conception interval (CCI) in the primiparous heifers and multiparous cows and in monitored and unassisted animals

		**AI per conception (n)**	**CCI (days)**
Parity	Primiparous (n = 360)	1.89 ± 1.35	140.7 ± 78.3
	Multiparous (n = 232)	2.08 ± 1.51	141.9 ± 75.3
Calving monitoring	Monitored animals (n = 120)	1.64 ± 0.98	115.0 ± 62.1
	Unassisted in calving barn (n = 120)	2.00 ± 1.39^*^	143.9 ± 83.4^†^
	Unassisted in dry zone (n = 352)	2.08 ± 1.54^†^	150.5 ± 77.7^¥^
Main effects	Parity	0.004	-
	Calving monitoring	0.021	0.001
	Parity x calving monitoring	0.005	-
	Mean square error	1.930	5764.78

^*^ = significantly different from the monitored animals at P < 0.05;

^†^ = significantly different from the monitored animals at P < 0.01;

^¥^ = significantly different from the monitored animals at P < 0.001.

The mean number of AI treatments per conception was also affected by the monitoring of calving condition and by parity (Table 3), but discontinuously; in fact, the number of inseminations of the HMO group was significantly fewer (*P* < 0.01) compared with the HUN group, but not in comparison to the HCB group. Within the multiparous groups, the monitored group demonstrated fewer than two inseminations per conception, compared with more than two inseminations per conception in the MCB (*P* < 0.01) and MUN groups.

## Discussion

The calving assistance assured by remote monitoring led to a reduction in stillbirths, FMR and uterine infections (Table 2). These results were related to the attenuation of dystocia’s sequelae, in fact, dystocia during delivery has a profound negative impact on dairy farming, reducing productivity and reproductive efficiency in the dams, increasing the odds of stillbirth and disease in calves and dams and resulting in both major treatment costs and animal welfare issues [3,28].

One of the possible solutions to attenuate the negative effects of dystocia is to increase calving surveillance [2], and application of a monitoring system appears to be a valuable tool in dairy calving management, as it reduces many of the costs associated with animal surveillance. Previously proposed protocols [8-15] allow the start of calving to be predicted within a window of several hours and also require frequent monitoring of cows. In contrast, the system employed in this study gave an accurate determination of the beginning of the second stage of labour. The device is inserted in the vaginal canal at the occurrence of liquefaction of the cervical mucus plug, limiting the time spent inside the body to a maximum of 96 h. However, in longer trials, during which the intravaginal device remained inside the vaginal canal for 2 consecutive weeks, no adverse effects were observed and the animals did not exhibit any discomfort or vaginal discharge. The device application allows assistance to be provided at the exact moment of delivery, improving the well-being of cows and calves during the calving process. Previous experience with equine alarm systems [18], which are maintained in site by suturing the device to the vulvar labia, have shown the occurrence of false alarms caused by the cow scratching in response to the suture. Moreover, the application of that system is cumbersome, requires the presence of a veterinary practitioner and would be disadvantageous economically for the dairy industry [29].

The present monitoring system, allowing the presence of specialised personnel during the delivery, reduces the incidence of stillbirth and the FMR (Table 3), which are known to be the most significant risk factors for clinical endometritis [30], thus leading also to a lower occurrence of uterine infections. Uterine contamination after parturition, which could cause significant decreases in productive and reproductive performances [31,32], are linked to the cleanliness of a farm, especially in the calving area, and to hygiene during assistance to parturitions [33-35]. Our results suggest that promptly administered obstetrical procedures are more relevant than the cleanliness of the calving barn. In fact, there were no significant differences in terms of uterine infections between the groups housed in the calving barn (HCB and MCB) and groups in the dry zone (HUN and MUN). Indirectly, this consideration allows us to define the importance of the calving assistance assured by the alarm device. In support of this, it was recently found that the duration of labour, particularly the 2nd and 3rd stages and trauma to the female genital tract with disruption of the physical barriers to infection, are more strongly implicated in the pathogenesis of uterine infections than environmental faecal contamination [30].

In our study, the monitored groups showed a shorter calving-to-conception period, which was more evident in heifers than in multiparous cows. In addition, the lower incidence of uterine infections is associated with a decrease in the mean number of AI treatments per conception. Uterine diseases are among the most expensive conditions challenging the dairy industry as lengthen the calving-to-conception interval [36-38]. Therefore the direct and positive consequences of calving assistance are the improvement in post-partum fertility, the reductions in the costs of treatments and AI and improved profits thanks to fewer days open.

In the present study, calving assistance to the monitored animals was assured by experienced PhD students, allowing an exact definition of the cause of dystocia, and this revealed that the major cause of dystocia was foetal malposition, both in primiparous and multiparous dams. Furthermore, 5% of the primiparous and 3.4% of the multiparous cows showed severe dystocia more often caused by foetal–pelvic disproportion, as highlighted by Arthur et al. [39].

The higher incidence of mild dystocia in the primiparous compared with multiparous cows was caused by a delay of more than 90 min in the second stage of labour; overall, approximately 67% of primiparous and 77% of pluriparous dams calved spontaneously. These findings were lower than those of Meyer et al. [40] who reported a higher rate of animals that did not need obstetrical assistance among both primiparous (71.4%) and multiparous (89.9%) cows, whereas Lombard et al. [23] showed that 48.8% of the calves born to primiparous dams and 70.6% of calves born to multiparous were delivered unassisted.

Severe dystocia is associated with stillbirths and calf death up to 30 days of age [21]. The single case of stillbirth in our study was associated with uterine torsion that required protracted obstetrical manipulations in a multiparous cow; this type of dystocia disrupted the cardiopulmonary system of the calf and all resuscitation efforts failed. However, timely and appropriate obstetrical assistance reduces the stillbirth rate, as shown in Table 2, where higher rates of calf death are evident in the unmonitored groups compared with monitored cows. The effects of dystocia on calves can be minimised through easily implemented on-farm procedures, such as the delivery of high quality colostrum immediately after birth [5,21]. Moreover, colostrum feeding is closely associated with animal welfare and its importance is highlighted by a law of the European Community (EU directive 97/2/CE) directing that the first milk should be administered within 6 h of birth in cattle.

## Conclusions

The use of a remote calving monitoring device facilitated the presence of trained personnel during calving leading to a reduction in the incidence of puerperal diseases, such as retention of foetal membranes and uterine infections and improving the potential future reproductive performance of the cows. Moreover, early intervention with appropriate obstetrical manipulations allowed the cause of dystocia to be resolved and colostrum to be administered immediately after birth, improving neonatal viability.

## Abbreviations

GSM: Global system for mobile communications; CP: Crude protein; NDF: Neutral detergent fibre; ADF: Acid detergent fibre; H: Heifers; M: Multiparous; HMO: Monitored heifers; HCB: Non-observed heifers in the calving barn; HUN: Unmonitored heifers; MMO: Monitored multiparous cows; MCB: Non-observed multiparous cows in the calving barn; MUN: Unmonitored multiparous cows; APGAR: Appearance, Pulse, Grimace, Activity, Respiration.

## Competing interests

All authors have applied for a patent relating to the device employed in the research. The authors have not received funding for the patent application and declare that they have no financial competing interests.

## Authors’ contributions

PC and PM carried out the experiments and contributed to literature searching and writing of the manuscript; SG contributed to literature searching, data analysis, and the writing of the manuscript; CM and PPB participated in the design and technological improvements of the device; MM carried out the design of the experiments and contributed to writing of the manuscript. All authors have read and approved the final manuscript.
